# Supplementary material for: Reducing Lung ATP Levels and Alleviating Asthmatic Airway Inflammation through Adeno-Associated Viral Vector-Mediated CD39 Expression
Source: Biomedicines. 2021 Jun 8;9(6):656. doi: 10.3390/biomedicines9060656 (PMC8228057; doi:10.3390/biomedicines9060656)
Supplement: Supplementary file 1 [file biomedicines-09-00656-s001.zip › biomedicines-1249326-supplementary.pdf]

Supplemental figures

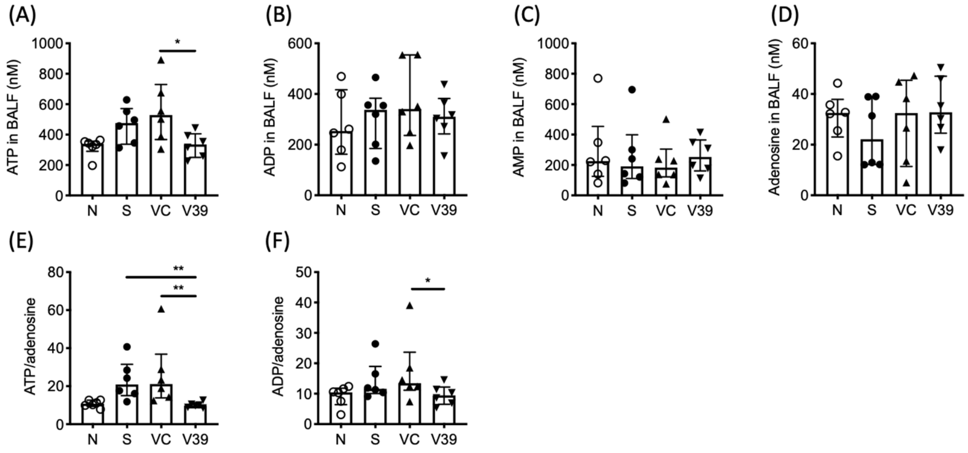

Figure S1. The ratios of ATP or ADP to adenosine were decreased in bronchoalveolar lavage fluid (BALF) of rAAV-CD39 treated mice. After the last OVA exposure, the lungs were lavaged by EDTA/PBS and the concentrations of (A) ATP, (B) ADP, (C) AMP and (D) adenosine were determined by LC-MRM. The ratios of (E) ATP to adenosine and (F) ADP to adenosine were calculated. Data are presented as median with interquartile range (*$p < 0.05$, **$p < 0.01$ in the comparison of V39 with S or VC).

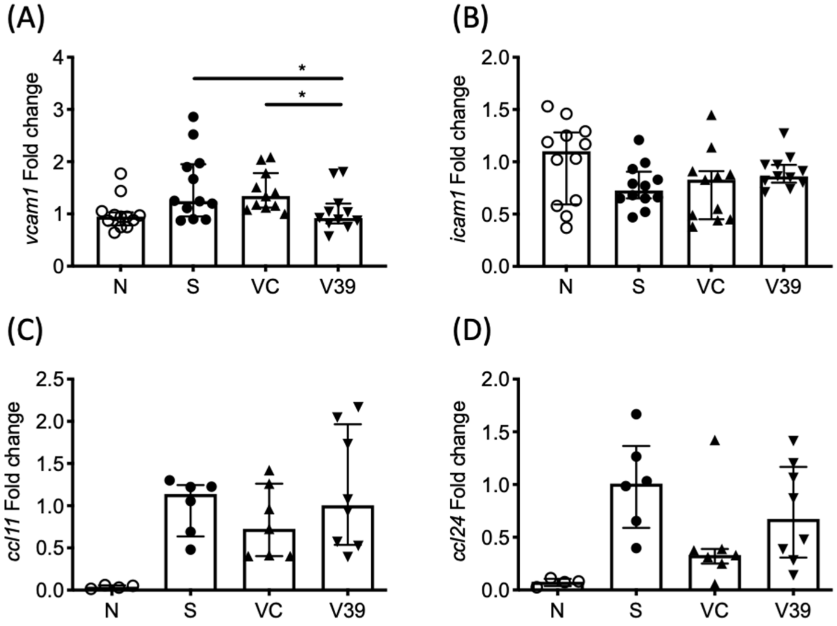

Figure S2. The RNA expression levels of adhesion molecule ICAM1 was reduced in rAAV-CD39 treated mice, but the expression of two eotaxins were not affected. RNA in lung tissues were extracted. The mRNA levels of (A) *vcam1*, (B) *icam1*, (C) *ccl11* and (D) *ccl24* to β-actin (*actb*) were analyzed by quantitative RT-PCR. Data are presented as median with interquartile range (**p* < 0.05 in the comparison of V39 with S or VC).

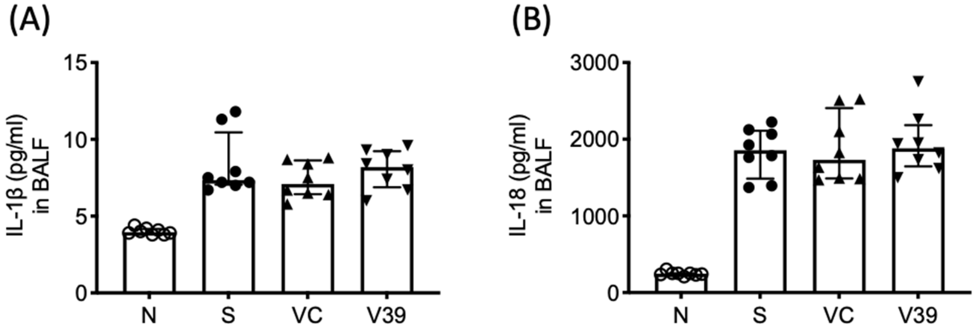

Figure S3. The concentrations of NLRP3-associated cytokines were not affected by the treatment of rAAV-CD39. After the last OVA exposure, the lungs were lavaged and the concentration of (A) IL-1β and (B) IL-18 in bronchoalveolar lavage fluid (BALF) were identified by ProcartaPlex. Data are presented as median with interquartile range.

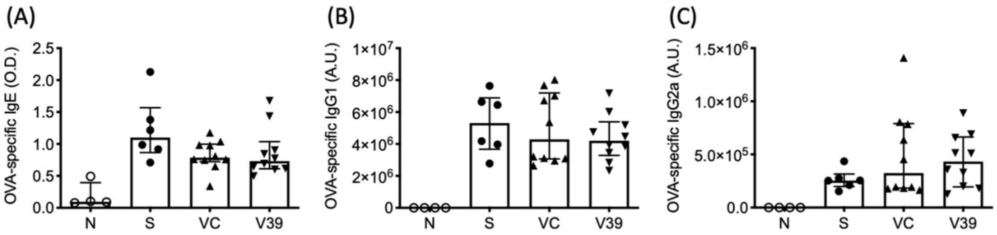

Figure S4. The levels of OAV-reactive antibodies were comparable in the OVA exposed groups. The serum of mice was collected 24 hours after the last OVA exposure, and the levels of (A) OVA-specific IgE, (B)OVA-specific IgG1, and (C) OVA-specific IgG2a were identified by ELISA. Data are presented as median with interquartile range.

Supplemental materials and methods

LC-MRM analysis of nucleotides

All of the reagents and solvents were acquired from Sigma-Aldrich (St. Louis, MO, USA), unless otherwise stated. The concentrations of analytes in BALF samples were determined according to a procedure slightly modified from previous research [1]. Briefly, to extract the metabolites in BALF samples, 150 µl of each sample was incubated with 225 µl of ice-cold methanol at -20°C for 20 min. After centrifugation at 18,000 g for 20 min at 4°C, the supernatant was collected, dried using a speed vacuum, and then reconstituted with 20 µl of 100 mM ammonium acetate. For each sample, we subjected 6 µl to LC-MRM analysis

The analysis was carried out on a high capacity spherical traps (HCT; Bruker) mass spectrometry coupled with a UPLC system (Waters) equipped with a porous graphitic carbon (PGC) column (Hypercarb, ThermoFisher Scientific; 100 mm X 2.1 mm, 5 µm). The mobile phases were: A1, 0.1 % formic acid (FA); B1, acetonitrile (ACN) in 0.2 % FA; A2, 0.1 % ammonium acetate adjusted to pH10 with ammonium hydroxide; B2, ACN. The flow rate was set to 300 µl/min. The analytes was separated with a 17 min gradient (0 min, 98% A2 and 2% B2; 2.0 min, 98% A2 and 2% B2; 4.0 min, 90% A2 and 10% B2; 13.0 min, 30% A2 and 70% B2; 17.0 min, 30% A2 and 70% B2). The column was then conditioned with 5% A1 and 95% B1 for 10 min and preconditioned with 98% A2 and 2% B2 for 7 min before next analysis.

For MS analysis, the positive mode was used and the selected transitions for each analyte is shown in Supplemental Table S3. The dry temperature was set to 200°C and the capillary voltage to 4000 V. Data analysis was performed using the Bruker Compass DataAnalysis (v4.2) software. The extracted ion chromatography (EIC) for each transition of each analyte was depicted and then smoothed using Gauss algorithm. Finally, the chromatic peak area of smoothed EIC were calculated. The response curve for each compound was constructed by subjecting varying amount of standard to LC-MRM analysis. The concentration of each analyte in each BALF sample was determined by comparing the peak area to that from respective response curve.

Quantification of OVA-specific antibodies

At the end of procedure, blood was collected and serum was prepared. OVA-specific IgE, IgG1 and IgG2a in serum were determined by ELISA as previously described [2].

Supplemental tables

Table S1. PCR primers

Primers	Nucleotide sequence (5'-3')		Ref.
actb	F	AGAGGGAAATCGTGCGTGAC	
	R	CAATAGTGATGACCTGGCCGT	
entpd1	F	CATCCAAGCATCACCAGACT	
	R	ATGATCTTGGCACCCTGGAA	[3]
ccl11	F	CTCACGGTCACTTCCTTCAC	
	R	TGCTGATATTCCCTCAGAGC	
ccl24	F	GCTGCACGTCCTTTATTTCC	
	R	CCCCTTTAGAAGGCTGGTTT	
icam1	F	GTGATGCTCAGGTATCCATCCA	
	R	CACAGTTCTCAAAGCACAGCG	
vcam1	F	CCAAATCCACGCTTGTGTTGA	
	R	GGAATGAGTAGACCTCCACCT	
entpd1 full length	F	AAGAATTCGCTGCCCCTTATGGAAG	
	R	AAGTCGACTACTGCCTCTTTCCAGA	

Table S2. Antibodies used in flow cytometry.

Markers	Dyes	clone	Company
CD3	FITC	145-2C11	BD bioscience
CD4	PE-Cy7	GK1.5	eBioscience
CD45	eFluor 506	30-F11	eBioscience
CD80	BV605	16-10A1	BD Horizon
CD86	Alexa 700	GL-1	BD PharMingen
FoxP3	APC	FJK-16S	eBioscience
I-A/I-E	eFluor 450	M5/114.15.2	eBioscience
IDO	PerCP-eFluor 710	mIDO-48	eBioscience
PDCA-1	PE-eFluor 610	eBio927	eBioscience

Table S3. Information for MS analysis.

ID	RT (min)	MS1	MS2
Adenosine	13.4	268	136
AMP	8.1	348	136
ADP	8.3	428	348
ATP	8.0	508	410

Supplemental references

1. Bustamante, S.; Gilchrist, R.B.; Richani, D. A sensitive method for the separation and quantification of low-level adenine nucleotides using porous graphitic carbon-based liquid chromatography and tandem mass spectrometry. *J Chromatogr B Analyt Technol Biomed Life Sci* **2017**, *1061-1062*, 445-451, doi:10.1016/j.jchromb.2017.07.044.
2. Wu, C.J.; Chen, L.C.; Huang, W.C.; Chuang, C.L.; Kuo, M.L. Alleviation of lung inflammatory responses by adeno-associated virus 2/9 vector carrying CC10 in OVA-sensitized mice. *Hum Gene Ther* **2013**, *24*, 48-57, doi:10.1089/hum.2012.039.
3. Neshat, S.; deVries, M.; Barajas-Espinosa, A.R.; Skeith, L.; Chisholm, S.P.; Lomax, A.E. Loss of purinergic vascular regulation in the colon during colitis is associated with upregulation of CD39. *Am J Physiol Gastrointest Liver Physiol* **2009**, *296*, G399-405, doi:10.1152/ajpgi.90450.2008.
